# Relationship among Chinese herb polysaccharide (CHP), gut microbiota, and chronic diarrhea and impact of CHP on chronic diarrhea

**DOI:** 10.1002/fsn3.3596

**Published:** 2023-08-06

**Authors:** Hong Xue, Chun‐Feng Mei, Feng‐Yun Wang, Xu‐Dong Tang

**Affiliations:** ^1^ Digestive Laboratory of Traditional Chinese Medicine Research Institute of Spleen and Stomach Diseases Xiyuan Hospital, China Academy of Chinese Medical Sciences Beijing China

**Keywords:** antidiarrheal effect, Chinese herb polysaccharides, chronic diarrhea, gut microbiota, small intestinal bacterial overgrowth (SIBO)

## Abstract

Chronic diarrhea, including diarrhea‐predominant irritable bowel syndrome (IBS‐D), osmotic diarrhea, bile acid diarrhea, and antibiotic‐associated diarrhea, is a common problem which is highly associated with disorders of the gut microbiota composition such as small intestinal bacterial overgrowth (SIBO) and so on. A growing number of studies have supported the view that Chinese herbal formula alleviates the symptoms of diarrhea by modulating the fecal microbiota. Chinese herbal polysaccharides (CHPs) are natural polymers composed of monosaccharides that are widely found in Chinese herbs and function as important active ingredients. Commensal gut microbiota has an extensive capacity to utilize CHPs and play a vital role in degrading polysaccharides into short‐chain fatty acids (SCFAs). Many CHPs, as prebiotics, have an antidiarrheal role to promote the growth of beneficial bacteria and inhibit the colonization of pathogenic bacteria. This review systematically summarizes the relationship among gut microbiota, chronic diarrhea, and CHPs as well as recent progress on the impacts of CHPs on the gut microbiota and recent advances on the possible role of CHPs in chronic diarrhea.

## Graphical abstract

This review systematically summarizes the relationship among intestinal flora, chronic diarrhea, and polysaccharides as well as recent progress on the impacts of Chinese herb polysaccharides on the gut microbiota and recent advances on the possible role of CHP in chronic diarrhea.

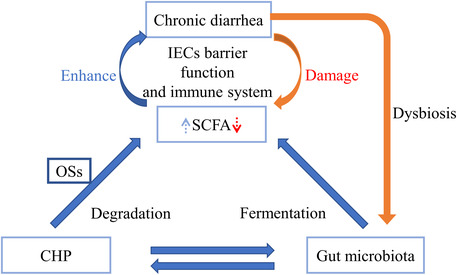

## 1 INTRODUCTION

Diarrhea is not a disease but a symptom of a debilitating and life‐threatening condition and may be associated with diverse conditions. Chronic diarrhea is usually associated with a number of noninfectious causes, including medications, irritable bowel syndrome (IBS), bile acid malabsorption, inflammatory bowel disease (IBD), hyperthyroidism, pancreatic insufficiency, and small bowel malabsorption (Arasaradnam et al., 2018). Chronic diarrhea is highly associated with dysregulation of intestinal homeostasis and gut microbiota composition (Li, Xia, et al., 2021). The gut microbiota is a complex microbiological system that is attracting increasing interest from the scientific community. The normal human gut microbiota comprises two major phyla, namely, Bacteroidetes and Firmicutes (Jandhyala, 2015). The majority of gut bacteria is nonpathogenic and cohabits with enterocytes in a symbiotic relationship, which further inhibits the colonization of most introduced pathogens and aids in nutrient absorption and physiological functions.

Although symptomatic treatment for diarrhea is practically necessary, particularly for chronic diarrhea, available drugs are limited and frequently not satisfactory. Numerous TCM formulas, such as Tong‐Xie‐Yao‐Fang (Zhang, Zheng, et al., 2022), Shen‐Ling‐Bai‐Zhu‐San (Ji et al., 2019), Fu‐Zi‐Li‐Zhong‐Tang (Zhen et al., 2021), Modified Renshen Wumei Decoction (Guan et al., 2021), and GeGen QinLian decoction (Li, Cui, et al., 2021) have been proven to be effective for curing or relieving diarrhea. Additionally, it has been demonstrated that amounts of Chinese herbs have antidiarrheal effects including *Panax ginseng C. A. Mey*. (Zhao et al., 2023), *Atractylodes lancea* (*Thunb*.) *DC*. (Qu et al., 2022), *Coptis chinensis Franch* (Xie et al., 2022), *Scutellaria baicalensis Georgi* (Kim et al., 2019), *Magnolia officinalis Rehd.et Wils*. (Xie et al., 2022), *Alisma orientale* (*Sam*.) *Juzep*. (Shu et al., 2016), *Pueraria lobata* (*Willd*.) *Ohwi* (Liu, Xu, et al., 2021), *Valeriana jatamansi Jones* (Ma et al., 2022), *Rhodiola crenulata* (*Hook. f. et Thoms*.) *H. Ohba* (L. Chen, Yu, et al., 2015), *Allium macrostemon Bge*. (Wu et al., 2019), *Euodia rutaecarpa* (*Juss*.) *Benth*. (Zhao et al., 2021), *Alpinia officinarum Hance* (Liang et al., 2013), *Santalum album L*. (Guo et al., 2014), *Engelhardia roxburghiana Wall*. (Wu et al., 2023), the fruit of Alpinia oxyphylla (*Alpinia oxyphylla Miq*.) (Wang et al., 2015), *Aconitum carmichaelii Debeaux* (Luan & Sun, 2014), *Astragalus membranaceus* (*Fisch*.) *Bge. var. mongholicus* (*Bge*.) *Hsiao* (Zhao et al., 2014), *Potentilla discolor Bge*. (Zhang et al., 2021), *Menispermum dauricum DC* (Su et al., 2016). In recent years, polysaccharides, as the most abundant biomacromolecules, obtained from many plants, animals, and microorganisms in nature have been found to offer advantages of safety, high therapeutic efficacy, nontoxicity, low cost, and good biocompatibility (Ho Do et al., 2021). Until now, more and more research have discovered that Chinese herb polysaccharides such as *Panax ginseng C. A. Mey*. (Li et al., 2019), *Schisandra chinensis* (*Turcz*.) *Baill*. (Qi, Chen, et al., 2019), *Astragalus membranaceus* (*Fisch*.) *Bge*. (Zhao et al., 2014), *Panax quinquefolium L*. (Ren et al., 2022), *Dioscorea opposita Thunb*. (Li et al., 2020; Zhang et al., 2019), *Poria cocos* (*Schw*.) *Wolf* (Xu et al., 2023), *Plantago asiatica L*. (Tian et al., 2020), *Polyporus umbellatus* (*Pers*.) *Fries* (Wang et al., 2000), *Rheum tanguticum Maxim. ex Balf*. (Liu et al., 2008), *Aconitum carmichaelii Debeaux* (Yang, Wu, et al., 2020), *Glycyrrhiza uralensis Fisch*. (Li, 2022), *Alpinia oxyphylla Miq*. fructus polysaccharide 3 (Luo et al., 2022), *Pogostemon cablin* (*Blanco*) *Benth*. (Chen, Luo, et al., 2020) can safely and effectively treat diarrhea. Recently, polysaccharides have been reported to exert modulatory effects on the gut microbiota, playing a critical role in protecting intestinal microecological homeostasis in the host (Ho Do et al., 2021). The gut microbiota is an important healthy “bridge” between polysaccharides and the human body (Song et al., 2021). On the one hand, polysaccharides, as the carbon source of intestinal microbial fermentation, fuel microbial growth and could serve as functional foods or prebiotics in preventing gut microbiota dysbiosis (Levy et al., 2017). On the other hand, intestinal microorganisms degrade polysaccharides into a variety of active metabolites (Levy et al., 2017), including monosaccharides, oligosaccharides, organic acids (such as ethanol, lactic acid, and succinic acid), and short‐chain fatty acids (such as acetic acid, propionic acid, and butyric acid) (Cheng et al., 2017). The interaction between CHPs and gut microbiota has become a hot research topic, and the biological activity of CHPs has an important influence on the regulation of gut microbiota.

Chronic diarrhea is highly associated with gut microbiota composition. Many reports have shown that gut microbiota dysbiosis, such as small intestinal bacterial overgrowth (SIBO), can cause chronic diarrhea, which also presents as abnormal gut microbiota. Polysaccharides derived from Chinese herbs produce a large number of oligosaccharides via degradation and fermentation by specific intestinal microbiota. Fermentation of polysaccharides and oligosaccharides (OSs) produces short‐chain fatty acids (SCFAs) and other metabolites that promote intestinal epithelial cell (IEC) barrier function and the immune system.

**FIGURE 1:**
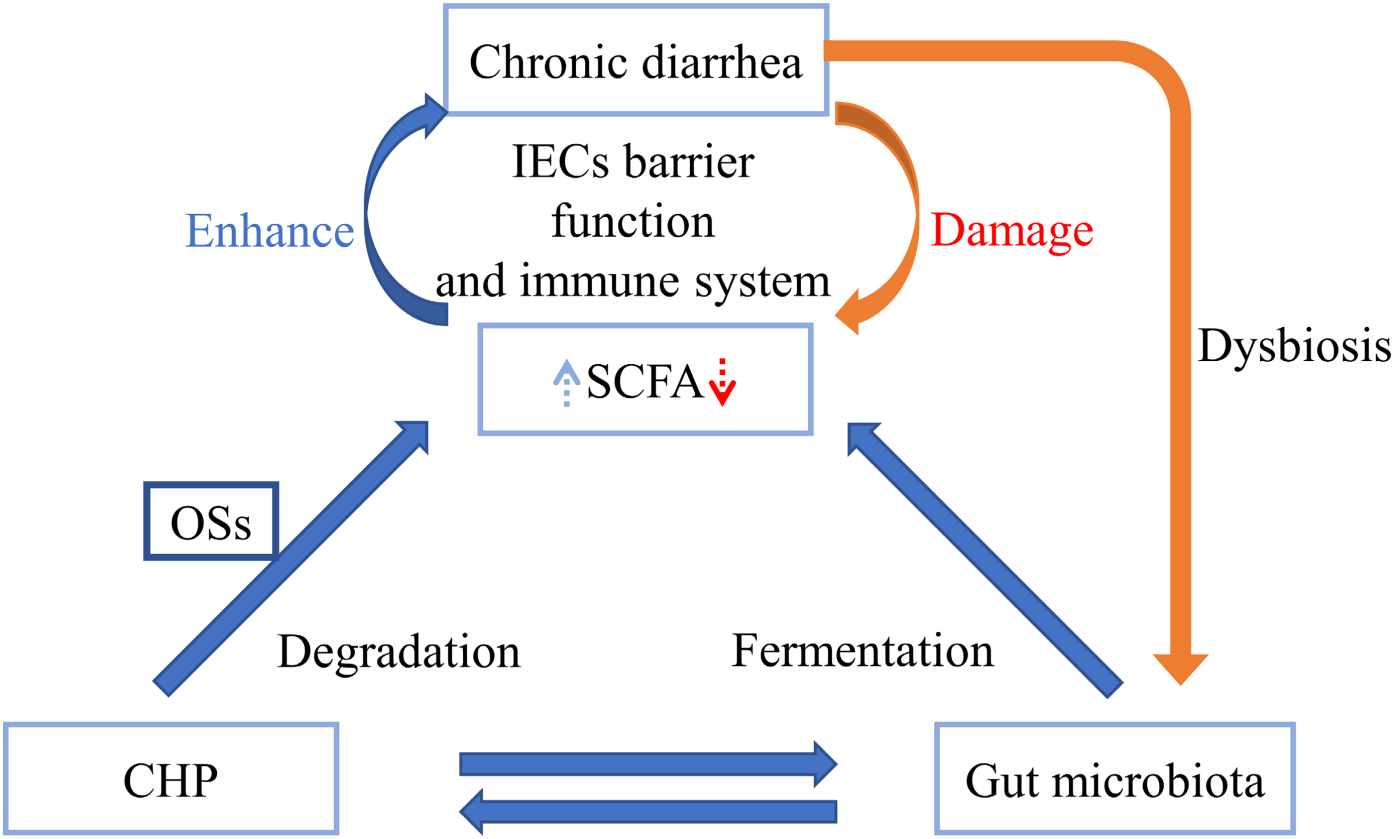
Relationship among Chinese herb polysaccharide (CHP), gut microbiota, and chronic diarrhea.

This review systematically summarizes the relationship among gut microbiota, chronic diarrhea, and CHPs as well as recent progress on the impacts of CHPs on the gut microbiota and recent advances on the possible role of CHPs in chronic diarrhea (Figure 1).

## 2 INTERACTION BETWEEN POLYSACCHARIDES AND GUT MICROBIOTA

### 2.1 Overview of Chinese herb polysaccharide

Polysaccharides are a class of natural macromolecular substances composed of 10 or more monosaccharides abundant in nature that widely originate from animals, plants, algae, and microorganisms (Yu et al., 2018). Traditional Chinese medicine (TCM) is one of the oldest medical systems worldwide, which is demonstrated to be rich in various polysaccharides, and has been the most important therapeutic method in China for thousands of years. The theory of homology between food and medicine has a long history in China. According to TCM theory, people are accustomed to calling substances that are both food and traditional Chinese medicine “medicine food homology (MFH) materials” (Yang, Su, & Chen, 2021). The natural products of MFH materials play an important role in human health and the application and development of the traditional Chinese medicine industry (Yang, Su, & Chen, 2021). Chinese herbal polysaccharides, as one of the most important active components in MFH materials, are mostly heteropolysaccharides that consist of different kinds of monosaccharides and have various biological activities (Wang et al., 2022) such as antitumor (Nie et al., 2017), immunologic enhancement (Cui et al., 2021), intestinal microenvironment regulation (Guo et al., 2021), and antioxidation (Chen, Zhao, et al., 2020) activities.

The structure of polysaccharides is more complex than that of proteins and DNAs, and the structure can be divided into primary, secondary, tertiary, and quaternary structures (Xie & Nie, 2010). The primary structure and chain conformation of polysaccharides are closely related to the construction of various functions (Zeng, Li, et al., 2019). The molecular weight of polysaccharides only represents the average distribution within a certain range of relative molecular masses, which is mainly expressed by the average molar mass (Mw) (Wang et al., 2022). The molecular weight of polysaccharides is an important feature affecting the therapeutic action of polysaccharides (Wang et al., 2022). The molecular weight shows a certain tendency of efficacy in an appropriate range, suggesting that higher or lower molecular weights might reduce the activity of CHPs (Wang et al., 2022). It is worth noting that CHPs with intestinal barrier protection have higher molecular weights (Błaszczyk et al., 2015), which may be because high molecular weight polysaccharides can maintain the integrity of the intestinal barrier structure by forming something similar to a sticky gel (Huo et al., 2022).

Chinese herbal bioactive polysaccharides can be extracted by a variety of modern techniques, including traditional water bath extraction, acid–base extraction, enzymolysis extraction, microwave‐assisted extraction, ultrasonic‐assisted extraction, ultrahigh pressure extraction, ultrasonic microwave‐assisted extraction, microwave‐assisted enzymatic extraction, and ultrasonic‐assisted enzymatic extraction (Su et al., 2021). Then, the crude polysaccharides were further purified because they contained many impurities, such as pigments, oligosaccharides, proteins, and inorganic small molecules. The purification techniques for the crude polysaccharides consisted of physical separation, chemical precipitation, and chromatographic purification. The proteins are removed using the Savage technique, the protease method, or the trichloroacetic acid method. Hydrogen peroxide solutions (H_2_O_2_) and microporous resins have become effective methods for pigment removal. After physical separation and chemical precipitation methods, column chromatography will purify polysaccharides to obtain high purity. The important point to note here is that purified polysaccharides undergo physiochemical and functional changes during preparation, especially differences in monosaccharides and molecular weights, resulting in multiple bioactivities.

A number of polysaccharides, including arabinose, fructose, fucose, galactose, glucose, mannose, rhamnose, ribose and xylose, arabinogalactans, and pectic acid arabinogalactan or pectin, are isolated from the roots and aerial parts of *Astragalus membranaceus* (*Fisch*.) *Bge*. (*A. membranaceus*) (Jin et al., 2014). Several components of ginseng polysaccharides have been identified and studied, including arabinogalactan, pectin, and acidic polysaccharides, which are mainly composed of monosaccharides such as L‐arabinose, D‐galactose, L‐rhamnose, D‐galacturonic acid, and D‐glucuronic acid (Wang et al., 2004). Up to 95% of the *Lycium barbarum L*. (LBP) consists of glycans including glucose, arabinose, galactose, mannose, xylose, rhamnose, and fucose (Wang, Chang, & Chen, 2009). Several components of *Angelica sinensis* (*Oliv*.) *Diels* (ASP) are mainly composed of monosaccharides such as glucose, mannose, galactose, rhamnose, arabinose, and xylose (Cao et al., 2006). In addition, some glucans are isolated and purified from *Angelica sinensis* (*Oliv*.) *Diels* (Cao et al., 2006). *Cordyceps sinensis* (*BerK*.) *Sacc*., a valuable traditional Chinese medicine, can be classified into two types according to their position in fungal cells, intracellular polysaccharides and extracellular polysaccharides (Wang, Wang, et al., 2009; Yan et al., 2014). *Ophiopogon japonicus* (*L. f*.) *Ker‐Gawl*. is a widely used traditional Chinese herbal medicine and is rich in polysaccharides composed of β‐fructose and a small amount of α‐glucose (Gong et al., 2017).

### 2.2 Impact of CHPs on the gut microbiota

Most herb polysaccharides cannot be digested directly by the human body after oral administration, owing to the lack of digestive enzymes that are encoded in the human genome (Gu & Shu, 2019). In contrast, gut microbes can encode a variety of carbohydrate‐active enzymes (CAZymes), including glycoside hydrolase, polysaccharide lyase, carbohydrate esterase, and glycosyl transferase (Lombard et al., 2014). These enzymes are required to digest most of our complex repertoire of polysaccharides. The presence of various types and quantities of CAZymes enables the gut microbiota to digest the carbohydrates in TCM via specific signaling pathways, thereby fermenting a variety of TCM and metabolizing polysaccharides in an efficient manner (Cao et al., 2014; Su et al., 2021; Terrapon et al., 2015). Meanwhile, CHPs function to reshape the composition of gut microbiota to restore pathological disorders (Chooi et al., 2019). It has been demonstrated that glycoside hydrolase, glycoside lyase, and glycoside esterase are involved in the process of glycolysis of *Ganoderma lucidum* (*Leyss. ex Fr*.) *Karst*. polysaccharide (Deng et al., 2006; Huang et al., 2016), *Taraxacum mongolicum Hand. Mazz*. polysaccharide (Huang et al., 2016; Shi & Zhang, 2016), *Astragalus aaronii (Eig) Zohary* polysaccharide (Huang et al., 2016; Zhang et al., 2014), *Cornus officinalis Sieb. et Zucc*. polysaccharide (Huang et al., 2016; Wang et al., 2014), *Morus alba L*. polysaccharide (Chen, Zhang, et al., 2015; Huang et al., 2016), and *Dioscorea opposita Thunb*. polysaccharides (Huang et al., 2016; Li et al., 2016). The microorganisms establish themselves in the gut through evolution of CAZymes capable of degrading specific polysaccharides (Martens et al., 2014). Accordingly, different types of bacteria have differing CAZyme content (El Kaoutari et al., 2013).

Polysaccharide utilization loci (PULs) are genomic loci that encode the CAZymes, glycan binding proteins, transporters, and sensors and regulators required for the binding, cleavage, and import of carbohydrates (Grondin et al., 2017). In general, degradation of polysaccharides is initiated by extracellular cleavage of the polysaccharide and is carried out by surface endo‐glycoside hydrolases. This produces large oligosaccharides that are then rapidly transported to the periplasm, where the oligosaccharide is broken down into simple monosaccharides and disaccharides (Cuskin et al., 2015). Despite efforts thus far, we have only begun to scrape the surface of CAZyme diversity within the human gut microbiota. Further research into how carbohydrates are partitioned within the gut microbiota will provide insights into how CHP may affect our overall health.

### 2.3 Degradation of polysaccharides by the human intestinal microbiota

**FIGURE 2:**
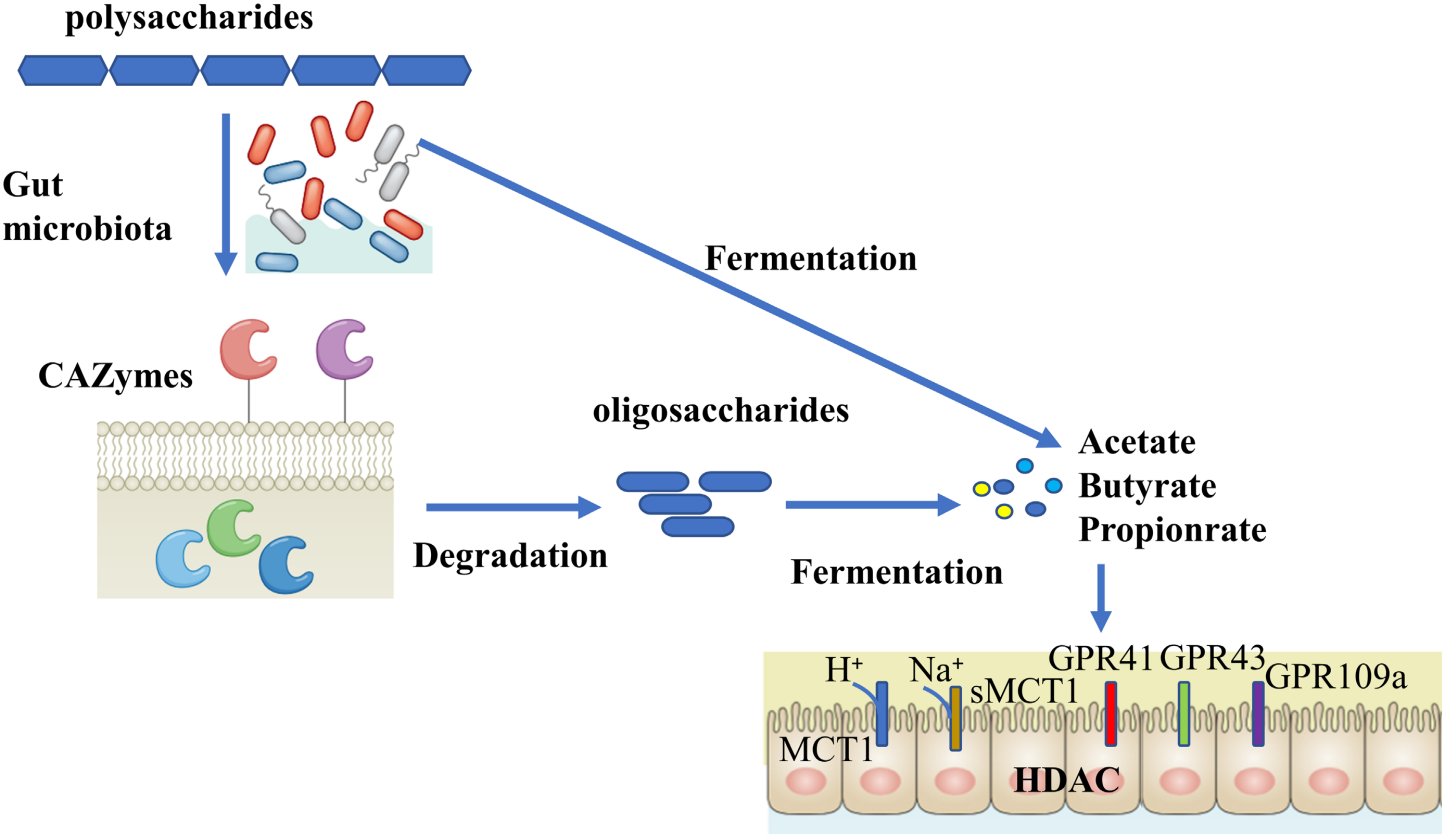
The degradation process of CHPs by the gut microbiota.

The biological activity of polysaccharides in the body is affected by their characteristics of digestion and glycolysis (Song et al., 2021). The mechanism of polysaccharide degradation in bacteria involves three main systems: the Sus‐like transport system, ABC‐transport system, and cellulosome‐like scaffolded enzyme system (Porter & Martens, 2017). Many CHPs, including *Bletilla striata* (*Thunb*.) *Reichb.f*. (Wang et al., 2023), *Dendrobium officinale Kimura et Migo* Xianhu 2 (Zhou et al., 2023), *Ophiopogon japonicus* (*L. f*.) *Ker‐Gawl*. (Wang, Wang, et al., 2019), *Lycium barbarum L*. (Ding et al., 2019), *Cordyceps sinensis* (*BerK*.) *Sacc*. militaris (Ying et al., 2020), Sijunzi decoction (Gao et al., 2018), *Sargassum pallidum* (*Turn*.) *C.Ag*. (Yuan et al., 2020), *Anemarrhena asphodeloides Bge*. (Chen et al., 2023), *Siraitia grosvenorii* (*Swingle*) *C. Jeffreyex A. M. Lu et Z. Y. Zhang* (Guo et al., 2022), and *Tremella fuciformis Berk*. (Dt et al., 2022), were supplied as the energy source to human bowel microorganisms in vitro fermentation could be mainly broken down, indicating that it was remarkably utilized by intestinal microbiota in human feces. Furthermore, these polysaccharides could remarkably enhance the abundance of beneficial microbiota and inhibit pathogenic bacteria, making them promising prebiotic candidates to prevent disease by promoting gut health. During the biochemical process of anaerobic digestion, polysaccharide chains are cut into shorter chains, then into simple sugars, and then fermented into intermediate metabolites such as formic acid, succinic acid, and lactic acid. The end products of this trophic chain are volatile fatty acids or short‐chain fatty acids (SCFAs), such as butyrate, acetate, and propionate (Wong et al., 2006) (Figure 2).

### 2.4 Gut microbiota involved in polysaccharide metabolism

Two major phyla dominate the human bowel microbiome kingdom, including the gram‐negative Bacteroidetes and the gram‐positive Firmicutes. Gram‐negative Bacteroidetes can degrade a relatively wide range of polysaccharides, and gram‐positive Firmicutes tend to metabolize a series of selected polysaccharides (Salyers et al., 1977). *Bifidobacteria longum* utilizes arabinoxylan oligosaccharides to generate acetate, which can be converted into butyrate by *Eubacterium rectale*, after which it is effective for organisms to remain healthy (Rivière et al., 2015). *B. thetaiotaomicron VPI‐5482*, the most studied gut bacterium, has been reported to be capable of utilizing various glycosaminoglycans (GAGs), including chondroitin sulfate (CS), dermatan sulfate (DS), hyaluronic acid (HA), and heparan sulfate (HS) (El Kaoutari et al., 2013; Ndeh et al., 2020; Rawat et al., 2022). *Streptococcus intermedius UNS 35*, an oral microbiota commensal, was the first Firmicute that showed GAG (CSA and CSC) utilization (Shain et al., 1996). *Bacteroides ovatus*, *B. thetaiotaomicron*, *and B*. *uniformis* ferment a particularly wide range of polysaccharides, and this versatility may help to explain their prevalence as dominant species in the colon (Qin et al., 2010; Tap et al., 2009; Walker et al., 2011). *B. cellulosilyticus* has been demonstrated to consume many carbohydrates, including Glu, Sac, Fru, Mal, Xyl, Gal, Ri, Mel, Man, Lac, and AG (Robert et al., 2007). Two families of Firmicutes, Lachnospiraceae and Ruminococcaceae, are particularly abundant in the human large intestine (Flint et al., 2012). Emerging evidence suggests that Firmicutes play key roles in polysaccharide degradation (Flint et al., 2012). *Ruminococcus* bromii‐related bacteria showed a much greater ability to degrade raw or boiled RS2 and RS3 starches (Ze et al., 2012). Among Lachnospiraceae, the ability to utilize starch has been reported for most members of the *Roseburia/Eubacterium* rectale group of butyrate‐producing bacteria (Aminov et al., 2006). *Roseburia* spp. produce a major, high molecular weight (> 180 kDa) amylase that is detectable by zymogram analysis (Ramsay et al., 2006). *Ruminococci* are gram‐positive, nonsporulating, and nonmotile cocci (Su et al., 2021). Three species degrade cellulose and metabolize cellobiose (Su et al., 2021). As described earlier, certain microorganisms can encode specialized CAZyme genes for polysaccharide degradation. In vivo, polysaccharides are usually attacked by various bacteria‐secreted enzymes. The gut microbiota can metabolize prebiotic polysaccharides and produce a wide range of primary and secondary metabolites.

### 2.5 Effect of polysaccharides on SCFAs, which play a key role in gut homeostasis

Certain gut bacteria degrade polysaccharides into SCFAs, mainly acetate, propionate, and butyrate. SCFAs, as an important energy source for intestinal microorganisms and hosts, play an important role in reducing intestinal pH, inhibiting pathogenic microorganisms, maintaining intestinal barrier integrity, and reducing the incidence of colon cancer (Tan et al., 2014). The effect of SCFAs on biological responses is based on two main signaling mechanisms, namely, the inhibition of histone deacetylases (HDACs) and signaling through G‐protein‐coupled receptors (GPCRs) (Cong et al., 2022). SCFAs can act as potent HDAC inhibitors (HDACis), and butyrate was found to be the most potent HDACi (Liu, Wang, et al., 2021), which might either act directly on HDACs by entering into the cells via transporters or indirectly through the activation of GPCRs (Liu, Wang, et al., 2021). SCFAs primarily increase gene transcription (Bilotta & Cong, 2019; Waldecker et al., 2008), regulate the host immune system and oxidative stress (Hamer et al., 2008), and enable cell cycle arrest, differentiation, and apoptosis at physiological concentrations by inhibiting GPCRs (Zeng, Umar, et al., 2019). HDACs are a class of enzymes that remove acetyl groups from ε‐N‐acetyl lysine on histones, thereby allowing the histones to wrap DNA more tightly (Liu, Wang, et al., 2021). Accumulating experiments have shown that SCFAs may participate in the activities of various organs and tissues of the human body through G‐protein‐coupled receptor 41 (GPR41), GPR43, and GPR109A (Tan et al., 2014). GPR41 is most sensitive to valeric acid, while GPR43 is most sensitive to propionic acid (Brown et al., 2003). SCFAs alter the secretion of glucagon‐like peptide 1 (GLP‐1) and YY peptide (PYY) by GPR43 and GPR41 (Christiansen et al., 2018). SCFAs can function as agonists for GPCRs that can regulate downstream signaling pathways, such as ERK/MAPK, JNK, p38, or Akt/PI3K, to control cell proliferation, differentiation, survival, and migration (Alfonzo‐Méndez et al., 2016; Gether, 2000; Venkatakrishnan et al., 2013). As HDACis, SCFAs can suppress the NF‐κB signaling pathway in mononuclear cells and neutrophils (Aoyama et al., 2010) and upregulate the expression of p21 Waf1/Cip1, IL‐8, VCAM‐1, and the transcription factor Foxp3 (Liu, Wang, et al., 2021). SCFAs can directly activate AMPK by increasing the ratio of AMP/ATP (den Besten et al., 2015, p. 1), and then AMPK can affect many downstream signaling pathways, such as mTOR, PGC‐1A, and FOX03 (He et al., 2020).

Many experiments have demonstrated that the concentration of SCFAs is increased after polysaccharides are degraded by the gut microbiota, but under the same fermentation conditions, their concentrations of total SCFAs are different. The experimental results clearly showed that there was a certain difference in the concentration of each SCFA produced by polysaccharides with different Mws, mainly because structural carbohydrates affect the fermentability of each microbiota in the gut. Recently, more and more studies started to be concerned about the effect of CHP on gut microbiota and SCFA. *Crataegus pinnatifida Bge*. polysaccharides could significantly increase the total SCFA concentrations in the gastrointestinal tract in dextran sulfate sodium (DSS)‐treated mice (Guo et al., 2021). *Atractylodes macrocephala Koidz*. polysaccharide (AC1) effectively increased significantly the abundance of *Lachnospiraceae_bacterium_A4*, *Bacteroides_vulgatus*, and *Prevotella_sp_CAG:891* and increased SCFA concentrations in constipated mice (Yang et al., 2023). *Ephedra intermedia Schrenk et C.A.Mey*. polysaccharide has a therapeutic effect in asthma‐like disease via regulation of gut microbiota and enhancing SCFA production (Liu et al., 2022). *Rehmannia glutinosa Libosch*. polysaccharides attenuate colitis by reshaping gut microbiota and increasing the content of short‐chain fatty acids (Lv et al., 2023). Li et al have demonstrated that the polysaccharide S‐3‐1 from Sijunzi decoction increased contents of acetic acid and total acid that were associated with its effects on the abundances of *Enterococcus*, *Sutterella*, *Butyricimonas*, and *Streptococcus* (Gao et al., 2018). *Paeonia suffruticosa Andr*. polysaccharide administration relieved hyperglycemia and renal injury by reconstructing gut microbiota and elevated the short‐chain fatty acid (SCFAs) contents (Zhang, Yang, et al., 2022). *Schisandra chinensis* polysaccharide could significantly regulate the imbalance of gut microbiota and increase the content of SCFAs on DSS‐induced ulcerative colitis (UC) in mice (Su et al., 2020). *Astragalus membranaceus* (*Fisch*.) *Bge*. polysaccharide (APS), a component derived from traditional Chinese medicine, could increase the SCFAs content by regulating the gut microbiota of chronic fatigue syndrome (Wei et al., 2023). *Dendrobium officinale Kimura et Migo* polysaccharide ameliorates polycystic ovary syndrome via increased butyrate and regulation of G‐protein‐coupled receptor 41 expression (Feng et al., 2022). Total polysaccharide from *Polygonum multiflorum Thunb*. (PS) could regulate short‐chain fatty acids (SCFAs) and their downstream signal protein molecules in the alleviation of insulin resistance (Bi et al., 2020). *Cistanche deserticola Y. C. Ma* polysaccharides could regulate the gut microbiota diversity as well as improve the production of short‐chain fatty acids (Fu et al., 2020). Polysaccharides from fermented *Asparagus officinalis* can be attributed to the upregulation of hepatic short‐chain fatty acid (SCFA) receptors GPR41 and GPR109A as well as intestinal SCFA production (Zhang et al., 2020). *Polygonatum odoratum* (*Mill*.) *Druce* polysaccharide (POP) administration also increased short‐chain fatty acids (SCFAs), including isobutyric acid, butyric acid, and valeric acid (Wang et al., 2018). With increasing research on polysaccharides and gut microbiota, the metabolic mechanisms of SCFAs have received increasing attention, especially the role of modulating gut microbiota to improve diseases related to glucose/lipid metabolism. Yang et al. (Yang, chang, et al., 2021) found that the isolation and purification of a homogeneous polysaccharide (LBP‐W) from *Lycium barbarum L*. increased gut microbiota diversity and specific genera, such as *Bacteroidetes* and *Lactobacillus*, which increased the content of SCFAs and significantly reduced hyperlipidemia‐induced obesity in mice. *Cyclocarya paliurus (Batalin) Iljinsk*. polysaccharides (CCPP) could alleviate type 2 diabetic symptoms by increasing SCFA‐producing bacteria, promoting the production of SCFAs, and upregulating SCFA‐GLP1/PYY‐associated sensory mediators (Yao et al., 2020). MDG‐1, a β‐D‐fructan polysaccharide extracted from the roots of Ophiopogon japonicas, could increase the contents of acetic acid and valeric acid, thus regulating inflammatory responses and hepatic lipid metabolism (Wang, Shi, et al., 2019). A water‐insoluble polysaccharide (WIP) isolated and identified from the sclerotium of *Poria cocos* (*Schw*.) *Wolf* elevated the level of butyrate in gut and increase butyrate‐producing bacteria *Lachnospiraceae*, *Clostridium* to regulate the lipid and glucose metabolism (Sun et al., 2019).

The polysaccharide was degraded by CAZymes encoded by the host gut microbiome to produce a large number of oligosaccharides. Fermentation of polysaccharides and oligosaccharides produces SCFAs and other metabolites. SCFAs can be easily absorbed by SCFA transporters, including MCT1 and sMCT1. Interactions of short‐chain fatty acids with intestinal epithelium based on histone deacetylases (HDACs) and G‐protein‐coupled receptors (GPCRs). During intestinal fermentation, polysaccharides, oligosaccharides, or metabolites such as SCFAs may promote the growth of certain intestinal bacteria, thus changing the composition of the intestinal microbiota.

## 3 GUT MICROBIOTA AND DIARRHEA

### 3.1 Introduction of normal gut microbiota

The intestinal tract of mammals hosts a high and diverse number of different microorganisms, including bacteria, fungi, protozoa, and viruses (Ryan et al., 2021), which constitute a huge and complex ecosystem in our gut. The colonic microbiome is dominated by mainly anaerobic bacteria, including thousands of species and millions of genes, distributed among the major phyla of Firmicutes (predominantly *Ruminococcaceae* and *Lachnospiraceae*), Bacteroidetes, Actinobacteria, Proteobacteria, and Verrucomicrobia (*Akkermansia*).

The gut microbial composition shows segmental differences across the GI tract, with a predominance of Firmicutes in the proximal colon and Bacteroidetes in the distal colon (Sekirov et al., 2010). Although everyone can harbor functional and distinctive variants of microbial composition due to early‐life events such as mode of delivery, type of feeding, and gender (Martin et al., 2016), three basic enterotypes, which are specifically characterized by clusters of bacteria, have been described in the gut microbiota in healthy adults, as stated by Arumugam et al (Arumugam et al., 2011). The microbiota has several roles, such as acting as a barrier against hexogen microbes, structural and metabolic functions (Hooper et al., 2001), and development and activation of the immune system (Belkaid & Hand, 2014; Kamada et al., 2013). These microbes produce key enzymes and metabolites that help to absorb essential nutrients and vitamins (Zhang et al., 2015) and are also important for the development and function of the mucosal immune system, which is required to provide a prompt and effective response to pathogens while remaining tolerant of harmless food antigens or other commensal species (Lloyd‐Price et al., 2016; Mishima & Sartor, 2020). Another essential role of this microbiota is pathobiont colonization resistance by filling distinct niches, exploiting oxygen, changing pH levels, outcompeting for nutrient resources, secreting transmitters, and inducing host immune activation (Buffie & Pamer, 2013; Sorbara & Pamer, 2019). Together, healthy resident microorganisms prevent invasive pathogens and maintain gut microbial ecosystems, which are important for the well‐being of the host.

### 3.2 Chronic diarrhea and gut microbiota dysbiosis

Chronic diarrhea is a complex and common problem in a gastroenterology clinic affecting up to 5% of adults and is defined as reduced stool consistency or an increase in the number of bowel movements for a period longer than 4 weeks (Schiller et al., 2017). Generally, diarrhea is a clinical manifestation of intestinal ion transport, and channel proteins and physical and chemical barriers are damaged, leading to disorders of intestinal epithelial water and electrolyte transport (Chu et al., 2020). In addition, diarrhea may be a symptom of many diseases, including functional diarrhea, irritable bowel syndrome (IBS) that is diarrhea predominant (IBS‐D) or postinfectious (PI‐IBS), celiac disease, and malabsorption syndromes such as lactose intolerance. It often leads to a reduction in quality of life, social functioning, malnutrition, and micronutrient deficiencies if without appropriate treatment. According to the pathophysiologic mechanism involved, chronic diarrhea can be divided into osmotic, secretory, inflammatory, malabsorptive, and secondary to dysmotility (Gómez‐Escudero & Remes‐Troche, 2021; Schiller et al., 2017). All these conditions have been associated directly or indirectly with gut microbiota alterations (or dysbiosis) (Scaldaferri et al., 2012).

The gut microbiota consists of major healthy resident microbiota providing beneficial effects for the host with multiple mechanisms (Kim & Jazwinski, 2018; Lloyd‐Price et al., 2016) and minor pathogenic species (Ghaisas et al., 2016; Sartor & Wu, 2017). These are presumed to be minor species and oppressed by beneficial ones in a healthy gut condition, although they increase and become activated when the intestinal environment undergoes changes, such as the usage of antibiotics, an altered diet and/or lifestyle, and exposure to severe enteritis (Richard & Sokol, 2019; Sartor & Wu, 2017). Such imbalance in the microbial community is termed “dysbiosis” and potentially has harmful effects on mucosal homeostasis (Drago et al., 2019; Holtmann et al., 2016).

### 3.3 Relationship between small intestinal bacterial overgrowth (SIBO) and chronic diarrhea

In healthy hosts, microorganism counts were shown to increase distally along the GI tract, with the proximal small bowel containing up to 10^3–5^ colony‐forming units of bacteria per milliliter (CFU/mL), whereas the colon could harbor up to 10^11^ CFU/ML (Rao & Bhagatwala, 2019; Sender et al., 2016). Small intestinal bacterial overgrowth (SIBO) is a condition in which the small bowel is colonized by colonic bacteria, formally defined by the presence of ≥10^5^ colony‐forming units per milliliter (CFU/mL) of jejunal aspirate by culture or a positive hydrogen lactulose or glucose breath test (Leite et al., 2020). SIBO is prevalent among multiethnic Asian adults with and without FGIDs (J. Zhao et al., 2010), especially for chronic diarrhea. Among the four subtypes of IBS, only IBS‐D is significantly associated with SIBO. One retrospective study showed that SIBO was more common in chronic nonspecific diarrhea (CNSD), including IBS‐D, than in other types of IBS and healthy controls (HCs) (Ghoshal et al., 2010).

SIBO and malabsorption have also been proposed as mechanisms underlying the development of diarrhea (Takakura & Pimentel, 2020). That SIBO could cause diarrhea in the absence of other clinical features of maldigestion or malabsorption (steatorrhea, malnutrition, and vitamin and nutrient deficiencies) was recognized more than half a century ago and noted to be especially prevalent among the elderly (Schiller, 2018). The subjects who had SIBO exhibited significantly elevated unconjugated bile salts, acetate, lactate, and formate compared with those without SIBO (Bala et al., 2006). The role of interactions between gut microbiota and bile acids in various aspects of SIBO has been evident for some time (Gracey, 1971); recent developments suggest that these interactions may play a key role in the pathogenesis of symptoms and gut dysfunction across the spectrum of SIBO (Bushyhead & Quigley, 2022). SIBO is associated with a myriad of symptoms, including but not limited to bloating, abdominal pain, nausea, constipation, and diarrhea, which share many symptoms with carbohydrate intolerance, making the clinical distinction of the disorders difficult, and further SIBO may represent an important reversible cause of carbohydrate intolerance (Perets et al., 2017). SIBO increases the likelihood of lactose intolerance in patients with chronic functional diarrhea (CFD) as a direct result of lactose fermentation in the small intestine, independent of oro‐cecal transit time and visceral sensitivity (Zhao et al., 2010).

### 3.4 Changes in gut microbiota in IBS‐D, osmotic diarrhea, antibiotic‐associated diarrhea, and bile acid diarrhea

Although scattered reports have shown that no significant difference was noted in the fecal microbiota between IBS‐D patients and controls in Thailand (Jandee et al., 2021), accumulating evidence has demonstrated that gut microbiota dysbiosis might be closely associated with the development of IBS symptoms, especially in IBS‐D patients (Zhuang et al., 2018). IBS‐D is characterized by an overall reduction in the microbial diversity of fecal microbiota and an increase in the ratio of Firmicutes/Bacteroidetes, a rough indicator of a microbial composition shift (Duan et al., 2019). The abundant phylum Firmicutes was significantly decreased and Bacteroidetes was increased in IBS‐D patients (Mei et al., 2021). A meta‐analysis found downregulation of bacterial colonization, including Lactobacillus, Bifidobacterium, and F. prausnitzii, in IBS patients, particularly in IBS‐D (Liu et al., 2017).

The fecal and mucus‐associated bacteria represent distinctive populations, with the latter more likely to influence the epithelium. Hou et al found that the composition of mucosa‐associated microbiota (MAM), not luminal microbiota (LM), was significantly different in IBS‐D patients compared to healthy controls (HCs), and there was a close relationship between the composition and function of MAM and clinical symptoms in IBS‐D patients (Yang, Hong, et al., 2020). A growing number of studies have supported the view that the number and composition of the microbial community in the feces and intestinal mucosa of IBS‐D patients are different. Ringel et al. showed a significant reduction in Bacteroides (Ringel & Ringel‐Kulka, 2015), while two studies showed an increase in this class, especially *Bacteroides vulgatus* and *Bacteroides fragilis*, both in mucosal and fecal samples, and *Bacteroides thetaiotaomicron* only in fecal samples (Ringel & Ringel‐Kulka, 2015; Shukla et al., 2015). One study indicated that IBS symptom severity was negatively associated with enterotypes enriched with Prevotella (Tap et al., 2017), while another study found that Prevotella was the most dominant genus in IBS‐D patients, while Bacteroides was more frequent in healthy subjects (Su et al., 2018). The difference between them may be caused by different original regions of patients and luminal microbiota, which tells us that the changes in composition and diversity of the gut microbiota in IBS‐D patients may be different, even leading to the opposite outcome (Zhan et al., 2020). Interestingly, Hansson et al found pathogenic Brachyspira species in the colonic epithelial surface or mucus layers in 40% of patients with IBS‐D compared with healthy individuals, suggesting a role for Brachyspira in the pathogenesis of IBS (Jabbar et al., 2021). All these evidence confirm the complexity in identifying a specific pathological profile of the intestinal microbiota to better orient the therapeutic approach (Altomare et al., 2021).

Osmotic diarrhea is the consequence of different diseases, including medication with different laxatives (Ziese & Suchodolski, 2021). Lactose intolerance is a disorder of intestinal digestion due to the decrease or complete loss of lactase phlorizin hydrolase (LPH) activity (Ziese & Suchodolski, 2021), leading to disability of lactose absorption and osmotic diarrhea. Recent research showed that lactose‐induced chronic diarrhea depleted the Lachnospiraceae NK4A136 group and Ruminococcaceae UCG‐ 005 and increased the relative abundance of *Lactobacillus, Escherichia Shigella*, and *Megamonas* in the cecal microbiota (Xue et al., 2020). Osmotic diarrhea decreased the richness of phylogeny and showed a strong tendency to balance individualized microbiota on the mucosa (Gorkiewicz et al., 2013). There are also research findings indicating that the microbiota of stool and mucosa in patients with osmotic diarrhea were significantly different (Gorkiewicz et al., 2013); for example, Firmicutes was the main bacteria in the mucous membrane, and Bacteroidetes was the main bacteria in the stool (Meng et al., 2020). A study of children under 5 years old with diarrhea caused by carbohydrate dyspepsia showed that the proportion of aerobic and facultative anaerobic microbes in samples of the infectious group induced by intestinal infections was much higher than that in the noninfectious group. The relative abundance of *Enterococcus* in the healthy control group was significantly higher than that in the noninfectious group and infectious group (Wen et al., 2018).

Antibiotic‐associated diarrhea (AAD) is defined as diarrhea that occurs in association with the administration of antibiotics and that cannot be explained otherwise (Bartlett, 2002). Diarrhea can occur during antibiotic treatment and up to 8 weeks after treatment cessation (McFarland, 2008). The prevalence of AAD among patients who receive antibiotics is approximately 5%–35% (McFarland, 2008). In a study of adult ambulatory patients receiving antibiotics for 5–10 days, the incidence of AAD was 17.5% (Beaugerie & Petit, 2004). The clinical course of AAD differed depending on whether *C. difficile* was involved, with most episodes of *non‐C. difficile* AAD being mild in severity and self‐limiting, lasting only a few days (Beaugerie et al., 2003). A meta‐analysis of bacteria related to antibiotic‐associated diarrhea in hospitalized patients showed that *Clostridioides* (*Clostridium*) *difficile*, *Clostridium perfringens, Klebsiella oxytoca*, and *Staphylococcus aureus* are the most prevalent among hospitalized patients with AAD in the world (Motamedi et al., 2021). The animal model of the AAD group showed a higher abundance of Proteobacteria and Actinobacteria. More importantly, *Lactobacillus* was significantly less abundant, while *Enterococcus* was significantly more abundant in the model group than in the control group (Xie et al., 2019). Furthermore, antibiotic treatment increased the abundance of *Citrobacter*, *Stenotrophomonas*, and *Glutamicibacter*, whereas antibiotics decreased the abundance of *Mycoplasma* and *Helicobacter* (Xie et al., 2019).

Bile acid diarrhea (BAD) is a common disorder that results from an increased loss of primary bile acids and can result in a change in microbiome, overlapping irritable bowel syndrome with diarrhea (IBS‐D). Gut microbiota is responsible for deconjugation, dehydrogenation, 7α‐dehydroxylation, and epimerization of primary BAs, producing secondary BAs in the gastrointestinal lumen and to mediate feedback control of BA synthesis (Sayin et al., 2013). Indeed, gut microbiota is a major regulator of BAs pool size and composition, which in turn regulate microbiota composition and richness and its characteristics. Bian et al. discovered that in a group of IBS‐D patients with excessive BA excretion (BA^+^IBS‐D), a specific gut microbiota characterized by enrichment of BA‐transforming Clostridia species is able to enhance total BAs excretion, which is mirrored by high levels of fecal BAs and serum 7‐hydroxy‐4‐cholesten‐3‐one (C4) (Zhao et al., n.d.). Another recent study showed that fecal bacterial diversity was reduced in patients with BAD, enriched a bacterial composition in 10 operational taxonomic units (OTUs) including members of the Lachnospiraceae family, Ruminococcaceae family, *Bifidobacterium longum*, *Prevotella copri*, *Akkermansia muciniphila*, and two members of the *Bacteroides genus* (Sagar et al., 2020) (Table 1).

**TABLE 1:** Altered gut microbial composition in IBS‐D, Osmotic diarrhea, and AAD.

Disease	Microbial shift	Reference
IBS‐D	*Lactobacillus ↓* *Bifidobacterium ↓* *F. prausnitzii* ↓	Liu et al., 2017
	*Firmicutes* ↓ *Fusobacteria* ↓ *Actinobacteria* ↓ *Proteobacteria*↑	Mei et al., 2021
	*Bacteroides*↓	Ringel & Ringel‐Kulka, 2015
	*Bacteroides vulgatus*↑ *Bacteroides fragilis*↑	Shukla et al., 2015
	*Prevotella*↓	Tap et al., 2017
	*Prevotella*↑	Su et al., 2018
	*Brachyspira*↑	Jabbar et al., 2021
Osmotic diarrhea	*Lactobacillus*↑ *Escherichia‐shigella* ↑*Megamonas*↑	Xue et al., 2020
	*Aerobic and facultative anaerobic microbes*↑ *Enterococcus*↓	Wen et al., 2018
AAD	*Clostridioides difficile*↑ Clostridium *perfringens*↑ *Klebsiella oxytoca* ↑*Staphylococcus aureus*↑	Motamedi et al., 2021
	*Proteobacteria*↑*Actinobacteria*↑ *Lactobacillus*↓ *Enterococcus*↑ *Citrobacter*↑*Stenotrophomonas*↑ *Glutamicibacter*↑ *Mycoplasma*↓ *Helicobacter*↓	Xie et al., 2019
BAD	*Lachnospiraceae*↑ *Ruminococcaceae*↑ *Bifidobacterium longum*↑ *Prevotella copri*↑ *Akkermansia muciniphila* ↑ *Bacteroides*↑	Sagar et al., 2020

### 3.5 FMT and chronic diarrhea

Fecal microbiota transplantation (FMT) is considered a promising therapeutic pathway for intestinal microbiota‐related diseases aiming to normalize or restore gut microbiota, by transferring stool from a healthy donor into the patient (Antushevich, 2020; Gupta & Khanna, 2017). FMT has been proven to be highly effective in refractory *Clostridium difficile* infectious enteritis, accounting for about one third of AAD cases. Zhang et al. have demonstrated that the use of FMT in critically ill patients with AAD got the good clinical outcomes without infectious complications (Dai et al., 2019).

In a randomized double‐blind trial, patients with IBS received anaerobic‐prepared donor FMT have improvements in fatigue and quality of life (El‐Salhy et al., 2020). Another study showed that FMT treatment can effectively alleviate the anxiety and depression behaviors of IBS‐D patients (Lin et al., 2021). In Cynomolgus Monkey model with chronic diarrhea, FMT can mitigate the appearance of diarrheal symptoms and restore the disturbance of gut bacteria by reducing the relative abundances of potential pathogens and increasing the beneficial bacterium (Tian et al., 2022). FMT is a longstanding Chinese treatment applied to gastrointestinal disease with gut dysbacteriosis; however, several questions remain to be answered, and further investigations are needed such as the optimal dose, ethics, administration route, and frequency of treatment.

## 4 INTERVENTION EFFECT OF CHP ON DIARRHEA BY MODULATION OF GUT MICROBIOTA

### 4.1 Traditional Chinese herbs improve chronic diarrhea

In recent years, traditional Chinese medicine has attracted extensive attention for its ability to treat gastrointestinal diseases due to its moderate treatment effect and low side effects. Traditional Chinese herbal formulas (CHFs), such as “Sijunzi Tang, Tongxieyaofang, Buzhongyiqi Tang, and Shenlingbaizhu Tang”, are widely used for chronic diarrhea therapy in Asia, and clinical studies have also found that CHF could significantly improve abdominal pain and diarrhea in D‐IBS and functional diarrhea patients (Gou et al., 2016; Jandhyala, 2015; Ji et al., 2022; Zhou et al., 2019). A growing number of studies have supported the view that Chinese herbal formulas alleviate the symptoms of diarrhea by modulating the fecal microbiota because they inevitably interact with the gut microbiota with poor oral bioavailability (Zu et al., 2018). Qinghua Zhixie decoction (QZD) consists of Fengfei Cao (*Pteris multifida Poir*), Dijin Cao (*Elsholtzia Ciliata Hyland*), Bugu Zhi (*Psoralea corylifolia*), Huanglian (*Coptis chinensis*), Muxiang (*Radix Aucklandiae*), Paojiang (*Baked ginger*), Baishao (*Radix paeoniae alba*), Fangfeng (*Radix Saposhnikoviae*), Cangzhu (*Rhizoma atractylodis*), Baizhu (*Rhizoma Atractylodis Macrocephalae*), and Xianhe Cao (*Agrimonia pilosa*) and can regulate the gut microbiota and reduce 5‐hydroxytryptamine and vasoactive intestinal polypeptides to improve diarrhea (Ji et al., 2022). Gegen Qinlian decoction (GD), as a classic prescription for diarrhea with IDHS, is composed of *Pueraria lobata* (*Willd*.) *Ohwi*, *Scutellaria baicalensis Georgi*, *Coptis chinensis Franch*., and *Glycyrrhiza uralensis Fisch*., which is reported to have the effect of stopping diarrhea and intestinal microbial regulation functions (Li, Zhang, et al., 2021). Xianglian Pill (XLP), a traditional Chinese pharmaceutical preparation for the treatment of gastrointestinal disease, may represent a promising candidate for the treatment of antibiotic‐associated diarrhea (AAD) by restoring intestinal microbiota and attenuating mucosal damage (Yang, Zhang, et al., 2021). Our recent study demonstrated that Shenlingbaizhu, a classical Chinese herbal formula, could alleviate lactose‐induced diarrhea by regulating the colonic luminal and mucosal microbiota and restoring intestinal ion transport (Xue et al., 2022).

In addition to TCM formulas, accumulating evidence has revealed that Chinese single herbs such as *Zingiber officinale Rosc*., fermented *Panax ginseng C. A. Mey*., deep‐fried *Atractylodes lancea*(*Thunb*.) *DC*. (DAR), berberine, and *Phellodendron chinense Schneid*. extract also have antidiarrheal properties by restoring the unbalanced gut microbiota (Jia et al., 2019; Ma et al., 2020; Qu et al., 2021; Shi et al., 2019).

### 4.2 Intervention mechanism of Chinese herbal polysaccharides on chronic diarrhea

As an increasing number of natural polysaccharides have been discovered as an important prebiotic resource to attenuate diarrhea by modulating the composition of the gut microbiota, different types of polysaccharides in dietary fiber used to treat diarrhea have been discussed in detail by Qi and Tester (Qi & Tester, 2019), and the recent advances of polysaccharides in the treatment of UC have been reviewed (Niu et al., 2021).

Chinese herbal polysaccharides (CHPs) are natural polymers composed of monosaccharides that are widely found in Chinese herbs and function as important active ingredients. American ginseng (*Panax quinquefolius L*.) is an herbal medicine with polysaccharides as its important active ingredient (Ren et al., 2022). The polysaccharides of *Panax quinquefolius L*. (WQP) can enhance the recovery of the intestinal structure in rats, reduce inflammatory cytokine levels, improve short‐chain fatty acid (SCFA) levels, promote recovery of the gut microbiota and intestinal mucosal barrier, and alleviate antibiotic‐related side effects such as diarrhea and microbiota dysbiosis caused by lincomycin hydrochloride (Ren et al., 2022). Polysaccharide derived from *Pueraria lobata* (*Willd*.) *Ohwi* (PPL) could relieve colonic pathological changes and gut microbiota dysbiosis caused by AAD (Chen, Liu, et al., 2020). The polysaccharides extracted from this two‐herb *Lycium barbarum L*. and *Astragalus membranaceus* (*Fisch*.) *Bge*. formula can protect against experimental ulcerative colitis, presumably by promoting the recovery of the intestinal barrier (Zhao et al., 2014). Qiweibaizhu powder crude polysaccharide helped to restore the diversity, relative abundance, and community structure of intestinal mucosal bacteria to a certain extent on AAD (Li et al., 2022). *Alpinia oxyphylla Miq*. fructus polysaccharide 3 (AOFP3) showed antioxidative activity in inhibiting PEDV reproduction. Therefore, AOFP3 was expected to be an anti‐PEDV drug. Pogostemon cablin (Blanco) Benth. is used to treat porcine epidemic diarrhea. *Pogostemon cablin* (*Blanco*) *Benth*. (PCPs) have antiviral activities against porcine epidemic diarrhea virus (PEDV) (Chen, Luo, et al., 2020). The water‐soluble *Panax ginseng C. A. Mey*. neutral polysaccharide (WGPN) could improve the gut microecology by recovering the ileum structure and improving the diversity and composition of the gut microbiota in AAD mice (Qi, Li, et al., 2019). *Acanthopanax senticosus* (*Rupr.etMaxim*.) *Harms* polysaccharides (ASPS) prevented LPS‐induced mucosal inflammatory damage and diarrhea by HIF‐1α/COX‐2 pathway downregulation (Fan et al., 2019). Recent study showed that polysaccharides from *Nemacystus decipiens* significantly relieve the symptom of mice with AAD, significantly increased the abundance of *Muribaculum*, *Lactobacillus*, and *Bifidobacterium* and decreased the abundance of *Enterobacter* and *Clostridioides* at genus level (Pan et al., 2023). *Poria cocos* (*Schw*.) *Wolf* polysaccharides (PCP) alleviated the symptoms of AAD mice by restoring seven characteristic species of intestinal tract, including the following species: *Parabacteroides distasonis*, *Akkermansia muciniphila*, *Clostridium saccharolyticum*, *Ruminococcus gnavus*, *Lactobacillus salivarius*, *Salmonella enterica*, *and Mucispirillum schaedleri* (Xu et al., 2023) (Table 2).

**TABLE 2:** Effect of CHPs on gut microbiota in chronic diarrhea.

Polysaccharides	Microbial shift	Disease	Reference
Shenlingbaizhusan Polysaccharide	*Akkermansia ↑* *Bifidobacterium ↑* *Blautia ↑* *Lactobacillus ↓* *Escherichia‐Shigella ↓* *Dubosiella ↓* *Albaculum ↑* *Bilophila ↑* *Coriobacteriaceae_UCG‐002↑* *Enterococcus ↓* *Helicobacter ↓* *Dubosiella ↓* *Collinsella ↓*	Lactose‐induced diarrhea	Xue et al., 2022
American ginseng polysaccharides	*Lactobacillus ↑ Bacteroides ↑* *Blautia ↓* *Coprococcus ↓*	Antibiotic‐associated Diarrhea	Ren et al., 2022
Pueraria lobata polysaccharides	*Oscillospira ↑* *Anaerotruncus ↑*	Antibiotic‐associated Diarrhea	R. Chen, Liu, et al., 2020
Lycium barbarum polysaccharides	*Bacteroidetes ↑* *Lactobacillus ↑* *Faecalibacterium ↑ Enterococcaceae ↓* *Enterobacteriaceae ↓*	Weaning diarrhea	Zhao et al., 2014
Astragalus polysaccharides	*Pseudomonas ↑* *Allobaculum ↓* *Coprococcus ↓*	Antibiotic‐associated Diarrhea	Zhao et al., 2014
Qiweibaizhu powder crude polysaccharide	*Proteobacteria ↓* *Lactobacillus ↓* *Enterococcus ↑*	Antibiotic‐associated Diarrhea	Li et al., 2022
Schisandra chinensis polysaccharides	*Blautia ↑* *Intestinibacter ↑* *Lachnospiraceae‐UCG‐008 ↑* *Ruminococcus‐1 ↓* *Ruminococcaceae‐UCG‐014 ↓* *Erysipelatoclostridium ↓*	Antibiotic‐associated Diarrhea	Qi, Li, et al., 2019
Ginseng neutral polysaccharide	*Lactobacillus ↓* *Bacteroides ↓* *Blautia ↓* *Coprococcus ↓*	Antibiotic‐associated Diarrhea	Qi, Chen, et al., 2019
Nemacystus decipiens polysaccharides	*Muribaculum ↑* *Lactobacillus ↑* *Bifidobacterium ↑* *Enterobacter ↓* *Clostridioides ↓*	Antibiotic‐associated Diarrhea	Pan et al., 2023
*Poria cocos* polysaccharides	*Parabacteroides distasonis ↓* *Akkermansia muciniphila ↓* *Clostridium saccharolyticum ↓* *Ruminococcus gnavus ↑* *Lactobacillus salivarius↓* *Salmonella enterica ↓* *Mucispirillum schaedleri ↓*	Antibiotic‐associated Diarrhea	Xu et al., 2023

## 5 CONCLUSION AND PERSPECTIVES

Increasing evidence has shown that alterations in microbial composition are closely associated with chronic diarrhea. Recent studies indicate that CHP may have an antidiarrheal role as an important energy source for gut microbiota. The interaction between gut microbiota and CHP has become a research hotspot. Notably, more clinical trials should be conducted to determine the medical effects of CHP on chronic diarrhea, which would open up new prospects in the fields of functional foods and pharmaceutics. Fermented products from these polysaccharides, especially SCFAs such as acetate, propionate, and butyrate, are bioactive molecules playing an important role by signal intestinal receptors or gut–brain neural circuits. How CHPs interact with the gut system needs to be deeply studied. Current human studies on adherent mucosal communities may be more valuable. In addition, the effect of bacterial metabolism, metabolic products, and functions of metabolites on the whole organism also need to be further studied. As a kind of prebiotic, CHPs are expected to maintain intestinal homeostasis by regulating the composition of the gut microbiota and provide new possibilities for the treatment of chronic diarrhea and the maintenance of host health.

## AUTHOR CONTRIBUTIONS

**Hong Xue:** Conceptualization (equal); investigation (equal); writing – original draft (equal); writing – review and editing (equal). **Chunfeng Mei:** Data curation (supporting); software (supporting). **Fengyun Wang:** Funding acquisition (equal); investigation (equal); supervision (equal). **Xudong Tang:** Funding acquisition (equal); supervision (equal).

## FUNDING INFORMATION

This research was funded by grants from the National Natural Science Foundation of China (Grant No.81973838).

## CONFLICT OF INTEREST STATEMENT

The authors declare that they have no competing interests.

## Data Availability

Not applicable.
